# ANCA-Negative Vasculitis in Eosinophilic Granulomatosis with Polyangiitis Complicated with Membranous Nephropathy: A Case Report and Brief Literature Review

**DOI:** 10.1155/2022/8110940

**Published:** 2022-05-06

**Authors:** Eri Kasama, Jun Ino, Fumika Iemura, Saeko Kumon, Mio Kodama, Keitaro Sato, Hitoshi Eizumi, Kosaku Nitta, Junichi Hoshino

**Affiliations:** ^1^Department of Nephrology, Kidney Center, Toda Central General Hospital, 1-19-3 Hon-cho, Toda City, Saitama 335-0023, Japan; ^2^Department of Nephrology, Tokyo Women's Medical University, 8-1 Kawada-cho, Shinjyuku-ku, Tokyo 162-0054, Japan

## Abstract

Renal involvement in eosinophilic granulomatosis with polyangiitis (EGPA) typically occurs in anti-neutrophil cytoplasmic autoantibody (ANCA)-positive cases presenting with rapidly progressive renal insufficiency and urinary abnormalities induced by primarily necrotizing crescentic glomerulonephritis (NCGN). Recently, ANCA-negative EGPA has also been reported to manifest with renal involvement, such as NCGN or non-NCGN, including membranous nephropathy (MN). Herein, we report a 70-year-old female who presented with purpura on the lower legs, upper limb numbness, renal dysfunction (eGFR, 20.5 ml/min/1.73 m^2^), and eosinophilia (eosinophils, 37,570/*μ*l). MPO-and PR3-ANCA were negative, and urinalysis revealed urine protein (0.63 g/day) but without red blood cells in the urine sediment. Thus, she was diagnosed with ANCA-negative EGPA with rapidly progressive renal dysfunction. A renal biopsy revealed vasculitis in the interlobular arteries without NCGN, with the vasculitis being complicated by MN. Micrograph findings on fluorescence immunostaining contained both primary and secondary characteristics of MN (dominance of IgG subclass 4 more than subclass 1 vs. negativity of PLA2R and THSD7A). After treatment with prednisolone, her eosinophil counts normalized, and renal dysfunction improved. Furthermore, urine protein did not increase above 1.0 g/day during the clinical course. This is a rare case of ANCA-negative EGPA presenting with acute renal dysfunction without NCGN and subclinical MN with unknown etiology. It is important to recognize that EGPA pathology varies widely throughout the disease course, and the clinical course of subclinical MN should be carefully assessed in further follow-ups.

## 1. Introduction

Eosinophilic granulomatosis with polyangiitis (EGPA) is a systemic vasculitis that is involved in anti-neutrophil cytoplasmic autoantibody (ANCA)-associated vasculitis (AAV). Although EGPA shares some common pathologies with AAV, microscopic polyangiitis, and granulomatosis with polyangiitis, EGPA has distinct clinical findings of severe asthma and eosinophilic sinusitis. Specifically, there is a significant increase in peripheral blood eosinophils that occurs several years before eosinophilic inflammation and vasculitis become apparent. ANCA occurs in approximately 40–60% of patients with EGPA [1]; however, the exact mechanism of the prevalence of EGPA and the role of ANCA in EGPA, particularly the association between ANCA and eosinophilic inflammation, remain unclear [2]. Moreover, ANCA-positive patients present with a greater frequency of renal insufficiency, sinusitis, and peripheral neuropathy than that in ANCA-negative patients [2].

Meanwhile, a typical renal histological finding in patients with EGPA is necrotizing crescentic glomerulonephritis (NCGN) that leads to rapid progressive renal deficiency. However, it was recently reported that 10% of EGPA was complicated with non-NCGN, such as membranous nephropathy (MN), of which most were ANCA negative [3]. Furthermore, MN complicated in EGPA has been previously reported for both ANCA-positive [4–6] and -negative [7, 8] cases. Although ANCA is recognized as a useful marker to differentiate the heterogeneous clinical spectrum of EGPA [9], it is difficult to clarify all clinical findings from ANCA results given the aforementioned exceptions.

We experienced a rare case of ANCA-negative EGPA, wherein rapid progressive renal insufficiency was caused by small vasculitis in the interlobular arteries rather than NCGN and was complicated with MN without nephrotic syndrome. We present herein the clinical course of this rare case, assessing the relationships among ANCA-negative EGPA, renal damage, and MN.

## 2. Case Presentation

A 70-year-old Japanese female, who was previously diagnosed with asthma 10 years ago and received steroid inhalation therapy from her primary doctor, developed general fatigue, appetite loss, numbness in the upper limb, and a red rash on the lower legs. Examination revealed a rapid decrease in renal function (creatinine [Cr], 1.78 mg/dL and estimated glomerular filtration rate [eGFR], 22.5 mL/min/1.73 m^2^ vs. Cr, 0.73 mg/dL and eGFR, 60.1 mL/min/1.73 m^2^ 4 months prior), so the patient was referred to our hospital. Blood examination on the day of admission revealed the following: peripheral eosinophilia (eosinophils, 37,570/*μ*L), upregulated inflammatory markers (white blood cells, 47,560/*μ*L; C-reactive protein, 1.0 mg/dL), renal dysfunction (Cr, 1.94 mg/dL; eGFR, 20.5 mL/min/1.73 m^2^), and negative PR3-ANCA and MPO-ANCA. Additionally, there were no prescriptions that could cause drug-induced eosinophilia. Urinalysis revealed urine protein (0.63 g/day) but without red blood cells in urine sediment. Although chest and brain computed tomography (CT) revealed no abnormal findings, CT of the sinuses demonstrated not only poor air-containing and hyper-absorptive areas predominantly in the left ethmoid sinus, which indicated rhinosinusitis, but also scattered polyps in the bilateral maxillary sinuses. The red rash on the lower legs and numbness in the upper limb were diagnosed as purpura by a dermatologist and as mononeuropathy by a neurologist, respectively. The patient met the new classification criteria for EGPA, recently published by the American College of Rheumatology/European Alliance of Association for Rheumatology (a minimum score of 6 points is required for the classification of EGPA) [10]. The patient scored 11 points: (1) bronchial asthma as an obstructive airway disease; +3 points, (2) Nasal polyps; +3 points, and (3) blood eosinophil count ≥1 × 10^9^/liter; +5 points.

**Figure 1:**
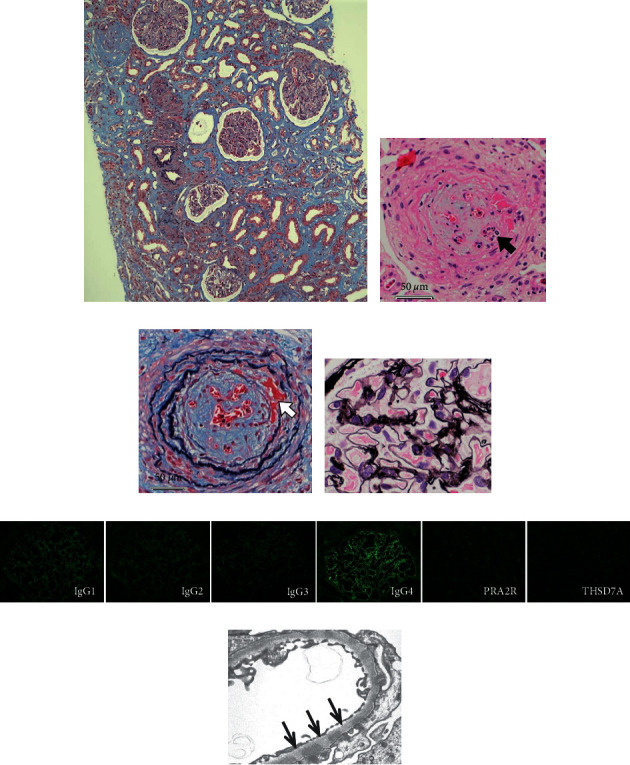
Histological findings of the renal specimen. Light microscopy image showing infiltration of inflammatory cells in the interstitial area and a severe degree of interstitial fibrosis and tubular atrophy (about 60%) but without granulomas ((a): PAS staining, original magnification ×100). Among the interlobular arteries, some show inflammatory cell infiltration in the lumen ((b): black arrow, hematoxylin and eosin staining, original magnification ×400), fibrin deposition in the vascular wall, and rupture of the elastic fibers ((c): white arrow, PAS staining, original magnification ×400). There are no significant abnormal findings in the glomerulus, including membrane changes ((d): periodic acid-methenamine silver staining, original magnification ×400). As shown in the micrograph of fluorescence immunostaining ((e): original magnification ×400), glomerular capillary deposits are dominant in IgG4. PLA2R and THSD7A are negative, suspicious of a secondary pathology. Electron microscopy image ((f): original magnification ×3000) showing subepithelial electron-dense deposits (EDDs) and a thickened basement membrane with spike formation (arrow). EDDs are limited to the subepithelium and are relatively uniform in size and distribution, which is not a finding of a secondary pathology.

After administration of prednisolone (PSL) 50 mg/day (1.0 mg/kg/day), the patient's general condition improved, eosinophils disappeared, and renal function showed slight recovery (Cr, 1.30 mg/dl; eGFR, 28.2 mL/min/1.73 m^2^). Percutaneous renal biopsy was performed 8 days after PSL administration, wherein light microscopy findings (Figures 1(a)–1(d)) showed 41 glomeruli without crescentic formation or apparent membrane changes. There was also moderate-to-severe infiltration of inflammatory cells into the interstitial lesion but without eosinophil infiltration and granuloma formation. Additionally, we observed inflammatory cell infiltration into the lumen in some interlobular arteries, fibrin deposition, necrotizing lesions on the vascular wall, and rupture of elastic fibers. Fluorescence immunostaining (Figure 1(e)) further showed dominant granular to linear glomerular capillary deposits in IgG4; however, PLA2R and THSD7A were negative. Electron microscopy (Figure 1(f)) showed subepithelial electron-dense deposits (EDDs) and a thickened basement membrane with spike formation. The EDDs were limited to the subepithelium and were relatively uniform in size and distribution. Considering these findings, two major histological diagnoses were made: (1) fibrinoid necrotizing vasculitis in microvessels and (2) early-stage MN.

After PSL was reduced to 40 mg/day, the patient was discharged and followed up at our outpatient clinic. She is currently well, and her renal function has recovered to almost equivalent to age-appropriate levels (Cr, 0.89 mg/dl; eGFR, 47.5 mL/min/1.73 m^2^). No abnormality in proteinuria was found 4 months prior (0.07 g/day) and at the latest visit in December 2021 (0.10 g/day). Symptoms including numbness and purpura improved after the administration of steroids, and no recurrence of these symptoms have been observed since. However, continuous inhalation therapy was necessary to stabilize asthma.

## 3. Discussion

We encountered a rare case of ANCA-negative EGPA that caused rapid progressive renal insufficiency and was complicated with MN without nephrotic syndrome. Histological findings revealed necrotizing vasculitis in the interlobular arteries and moderate-to-severe infiltration of inflammatory cells (not eosinophils) into the interstitial lesion as the EGPA pathology. Meanwhile, MN pathology was characterized by subepithelial EDDs and a thickened basement membrane with spike formations in the glomerular capillaries, as observed on electron microscopy.

The difference in the clinical findings of EGPA is reported to be related to two types of tissue damage linked to the diversity of ANCA: ANCA-mediated small vessel inflammation in ANCA-positive cases or eosinophilic inflammation in ANCA-negative cases [2]. In particular, the kidney is recognized as a representative target organ in ANCA-positive EGPA, with 80% of EGPA patients with renal involvement being ANCA positive [11]. A previous multicenter study also reported that rapid progressive renal insufficiency (52%) and isolated urinary tract abnormalities (48%) were two major renal clinical findings and that, as an important histological sign supporting renal insufficiency, NCGN presented in ANCA-positive EGPA patients [12]. Therefore, the rare feature of our case is acute renal impairment in ANCA-negative EGPA, which was likely caused by necrotizing vasculitis of the interlobular arteries rather than the typical NCGN. Other reports of ANCA-negative EGPA with renal involvement that were evaluated with renal biopsy also showed a heterogeneous clinical picture of vasculitis. Notably, one case reported pauci-immune crescentic glomerulonephritis and pulmonary hemorrhage [13], and another case reported isolated proteinuria with glomerular angionecrosis and eosinophil infiltration complicated by intracardiac thrombus [14].

Clinical diversity in patients with ANCA-negative EGPA might be associated with the pathological diversity that could occur over the long-term process of EGPA development. Originally, EGPA development involves three common phases [15]: prodromal localized inflammation by an unknown antigen with a genetic component, eosinophilic inflammation triggered by Th2-related cytokines [16], and ANCA-induced vasculitis [17]. Considering the transition of these phases, EGPA does not always show a consistent clinical picture within each phase. Therefore, we speculated that ANCA-negative vasculitis could occur as a clinical picture that is different from the completed picture of phase 3. This means that ANCA in tissues could cause vasculitis even if the circulating ANCA is not yet at an identifiable level. Although the precise mechanisms leading to the occurrence of ANCA-negative vasculitis remain unclear, ANCA-negative pauci-immune NCGN appears to be a part of a systemic vasculitis disease process [18]. Therefore, depending on the timing of the examination and diagnosis in the long-term course, EGPA could present with varying pathophysiological findings, including the extent of ANCA involvement.

On the other hand, a recent report of 63 renal biopsy-proven EGPA cases revealed that eosinophilic infiltration could contribute to renal involvement in EGPA [3]. In the present case, there was no significant infiltration of eosinophils into the interstitial tissues, which made it difficult to determine the connection between renal dysfunction and eosinophilic inflammation. However, the absence of eosinophilic inflammation in the renal tissue in our case could be explained by the patient's good response to steroids for EGPA, thereby resolving eosinophil infiltration after steroid treatment [9]. Hence, rapid progressive renal insufficiency without NCGN in the present case was deemed to have occurred due to necrotizing vasculitis in the interlobular arteries and, possibly, eosinophil infiltration.

The unique features of MN in this case included the absence of nephrotic syndrome, which is reported to occur in 70–80% of MN patients [19], and histological findings of both primary and secondary characteristics. If the present case was primary MN that was accidentally identified in subclinical nephrotic syndrome, old age would be the most likely trigger, and further examinations and treatments for proteinuria should be considered in the future. Whereas, in a case of secondary MN complicated with EGPA, a relationship beyond accidental coexistence is most likely, based on the following reports: MN could occur secondary to systemic diseases, such as autoimmune diseases [20]; 10% of EGPA was identified to be complicated with MN, of which most were ANCA-negative [3]; both conditions may share a genetic background of a common HLA allele; and both pathological conditions are similarly mediated by Th2 inflammatory response [21]. However, if the present case was a secondary MN related to EGPA, it makes sense to clinically and pathologically recognize large amounts of urinary protein and more advanced MN findings, respectively, in line with the timing of EGPA onset due to its increased activity. Similarly, although the dominance of IgG4 staining, which is characteristic of primary MN [22], could occur in advanced-stage secondary MN [23], the fact that our case was in the early stage without spike formation prompted us to consider primary MN. Additionally, although recent studies have revealed the distinct genetic predisposition of primary MN patients [24] and the involvement of self-antigens PLA2R and THSD7A in the development of primary MN [25, 26], primary MN with negative results for both self-antigens is not uncommon, with an incidence of up to 40% in Japan [27]. A previous study also reported that self-antigens other than PLA2R and THSD7A were possibly involved in MN [23], further supporting that MN was likely to be primary in our case. Furthermore, the localization pattern of EDDs to the subepithelium was also suggestive of primary MN [28].

We compared the clinicopathological features described in the six previous case reports of EGPA complicated with MN, including both ANCA-positive [4–6] and negative cases [7, 8], with our case (Table 1). We found that those with rapidly progressing renal deficiency demonstrated NCGN histologically, except for our case, and that renal function was recovered in almost all cases. Contrarily, those without renal dysfunction had no NCGN. Although eosinophilic infiltration was 50%, the close association between eosinophilic infiltration and MN, as previously reported [3], remained unclear, owing to scarce information on histological findings. All cases also demonstrated nephrotic syndrome, except for our case, and showed good responsiveness to therapies, reaching complete or partial remission. Moreover, all clinicopathological and prognostic factors appeared to be independent of ANCA positivity. A notable difference we found was that the present patient was older than the six previous ones. Thus, it was considered that the accidental finding of age-related MN complication and age-related effects on immune response were responsible for the different clinical findings.

In the present case, we determined that active treatment was not required for subclinical MN. Although our EGPA case presented with renal impairment, for which immunosuppressive agents are recommended based on FFS scoring [29], the consent for these therapies was not obtained by the family due to the patient's age. Furthermore, 2 weeks after azathioprine was induced for maintaining remission, cytopenia, which was thought to be due to azathioprine-induced myelosuppression, occurred. As a result, steroid monotherapy for EGPA was conducted, and the patient's clinical course was uneventful and improved without any particular symptoms in our outpatient management.

## 4. Conclusions

In EGPA, ANCA positivity, clinical, and histological findings may not appear consistently with each other as these could depend on the timing of diagnosis during the long-term process of EGPA development. It also remains unclear whether the etiology of the present MN was primary or secondary. Therefore, it is important to pay close attention to the critical pathologies of MN and the possibility of reactivation of EGPA in the future.

## Figures and Tables

**Table 1:** Cases of EGPA complicated with MN.

Author	Age/sex	Before treatment renal function (Cre mg/dL)	Other organ comorbidities	ANCA	NCGN/EOS	IF	Treatments	After treatments Cre (mg/dL)/proteinuria
(year)		Proteinuria						Other organ comorbidities

Ayuzawa (2012)	65/F	Decreased (Cre 0.9)	Purpura, neuropathy, pneumonia	Negative	+/+	IgG1 > 4	mPSL 1 g × 3 days PSL 40 mg (initial dose)	Cre 0.6/CR
		9.9 g/gCre						Improved
Ram (2014)	37/M	Decreased (Cre 2.3)	Respiratory syndromes, subcutaneous nodules, arthritis	Positive	+/ND	IgG	mPSL 15 mg/kg/day × 3 days PSL 0.5 mg/kg/day (initial dose) IVCY × 9 times, AZA 2 mg/kg/day	Cre 1.2/CR
		8.7 g/day						ND
Mahmood (2019)	63/F	Preserved (Cre ND)	Purpura, neuropathy, pneumonia	Positive	−/ND	IgG	Rituximab (regimen unknown)	ND/PR
		8.0 g/gCre						Improved
Kondo (2020)	50/F	Preserved (Cre 0.54)	IgG4-related disease, neuropathy	Negative	−/−	IgG4 > 1	PSL 0.8 mg/kg/day (initial dose) CsA 1.5 mg/kg/day (initial dose) MZB 3.0 mg/kg/day, IVIg × 3 times	ND/CR
		8.4 g/gCre						Neuropathy did not improve
Zhu (2019)	50/M	Preserved (Cre 0.87)	Purpura, pneumonia	Positive	−/+	IgG4, PLA2R	PSL 60 mg IVCY × 9 times	ND/CR
		6.4 g/gCre						ND
Our case	70/F	Decreased (Cre 1.94) 0.63 g/gCre	Purpura, neuropathy,	Negative	−/−	IgG4 > 1	mPSL 0.5 g × 3 days PSL 40 mg (initial dose)	Cre 0.86/CR
								Improved

ANCA, anti-neutrophil cytoplasmic antigen; AZA, azathioprine; Cre, creatinine; CsA, cyclosporine; CR, complete remission; EOS, eosinophilic infiltration; IF, immunofluorescence staining; IVCY, intravenous cyclophosphamide; IVIg, intravenous immunoglobulin; mPSL, methylprednisolone; MZB, mizoribine; ND, no data; NCGN, necrotizing crescentic glomerulonephritis; PR, partial remission; PSL, prednisolone.

## Data Availability

Due to confidentiality rules, the patient's medical data (beyond those directly reported in this manuscript) are not available for external dissemination.
